# Methodology of evaluation of morphology of the spine and the trunk in idiopathic scoliosis and other spinal deformities - 6^th ^SOSORT consensus paper

**DOI:** 10.1186/1748-7161-4-26

**Published:** 2009-11-26

**Authors:** Tomasz Kotwicki, Stefano Negrini, Theodoros B Grivas, Manuel Rigo, Toru Maruyama, Jacek Durmala, Fabio Zaina, Members of the international Society on Scoliosis Orthopaedic and Rehabilitation Treatment (SOSORT)

**Affiliations:** 1 Department of Pediatric Orthopaedics, University of Medical Sciences, Poznan, Poland; 2 ISICO (Italian Scientific Spine Institute), Milan, Italy; 3 Department of Orthopaedics and Traumatology, "Tzanio" General Hospital of Piraeus, Piraeus, Greece; 4 Instituto Èlena Salvá, Barcelona, Spain; 5 Department of Orthopaedic Surgery, Saitama Medical Center, Saitama Medical University, Saitama, Japan; 6 Department of Rehabilitation, Medical University of Silesia, Katowice, Poland

## Abstract

**Background:**

Comprehensive evaluation of the morphology of the spine and of the whole body is essential in order to correctly manage patients suffering from progressive idiopathic scoliosis. Although methodology of clinical and radiological examination is well described in manuals of orthopaedics, there is deficit of data which clinical and radiological parameters are considered in everyday practise. Recently, an increasing tendency to extend scoliosis examination beyond the measure of the Cobb angle can be observed, reflecting a more patient-oriented approach. Such evaluation often involves surface parameters, aesthetics, function and quality of life.

**Aim of the study:**

To investigate current recommendations of experts on methodology of evaluation of the patient with spinal deformity, essentially idiopathic scoliosis.

**Methods:**

Structured Delphi procedure for collecting and processing knowledge from a group of experts with a series of questionnaires and controlled opinion feedback was performed. Experience and opinions of the professionals - physicians and physiotherapists managing scoliosis patients - were studied. According to Delphi method a Meeting Questionnaire (MQ) has been developed, resulting from a preliminary Pre-Meeting Questionnaire (PMQ) which had been previously discussed and approved on line. The MQ was circulated among the SOSORT experts during Consensus Session on "Measurements" which took place at the Annual Meeting of the Society, totally 23 panellists being engaged. Clinical, radiological and surface topography parameters were checked for agreement.

**Results:**

90% agreement or more was reached in 35 items and superior than 75% agreement was reached in further 25 items. An evaluation form was proposed to be used by clinicians and researchers.

**Conclusion:**

The consensus was reached on evaluation of the morphology of the patient with idiopathic scoliosis, comprising clinical, radiological and, to less extend, surface topography assessment. Considering the variety of parameters indicated by the panellists, the Cobb angle, yet the gold standard, can be seen neither as the unique nor the only decisive parameter in the management of patients with idiopathic scoliosis.

## Background

Before a therapeutic intervention of any clinical condition, careful evaluation of the disorder should be performed. In the case of idiopathic scoliosis, the deformation of the body is usually (and superficially) identified with the disease itself, because the underlying pathomechanisms remain obscure [1]. Thus, the assessment of the body deformity remains the principal way of evaluation of the disease [2]. This assessment is essential for the management of idiopathic scoliosis in childhood and adolescence [3], as well as for the evaluation of the treatment outcome [4].

A typical clinical examination consists of inspection, palpation, percussion and auscultation, the two latter not involved in scoliosis patients. Moreover, the appraisal of the patient's morphology can be made both for static and dynamic conditions (during gait). Traditionally, the clinical exam of scoliosis is static and includes the assessment of the asymmetries of the shoulders, scapulae, flanks, hips, the plumb line exam, trunk imbalance, disturbances in sagittal curvatures and rotational phenomena (rib prominence, lumbar prominence), [3]. The findings are usually noted in a descriptive manner, for example: "protruding left hip", "right shoulder higher than the left one", "thoracic kyphosis slightly reduced" etc. Such records reveal useless in providing data suitable for scientific analysis because of its qualitative nature.

Although every manual of orthopaedics tends to present a comprehensive view of the clinical and radiological examination of scoliosis, there is a deficiency of data about the methods which are currently used in everyday practise by the professionals and about the parameters considered important. An increasing tendency to extend the examination beyond the assessment of the Cobb angle is observed, reflecting a more patient-oriented approach to spinal deformities [5]. Such assessment may include surface parameters, aesthetics, function and quality of life.

Subjective observation made by SOSORT members on insufficient skills and knowledge of medical students and residents concerning scoliosis examination was another impulse to undertake this study.

The purpose of this study was to conduct a systematic analysis [6] of the experts' opinion and experience related to the methodology of evaluation of the morphology of patients suffering from idiopathic scoliosis.

## Methods

The typical steps [7] of the Delphi method [6] were performed. After the topic of the consensus was defined, the group of experts in scoliosis treatment comprising SOSORT members was constructed, based on the professionals participating to previous consensus procedures. The first questionnaire was prepared, checked by the authors and distributed via e-mails among the SOSORT Board Members. The responses were analyzed and the Pre-Meeting Questionnaire (PMQ) was created (Additional File 1), then distributed electronically among the participants. The answers to PMQ were collected and analyzed, then the Meeting Questionnaire (MQ) was prepared (Additional File 2). During the SOSORT Annual Meeting the Consensus Session was organized, chaired by the first author (T.K.). Each item of the MQ was illustrated with PowerPoint presentation prepared by the Chairman, moreover participants could make short presentations concerning the particular items under discussion. The answers to the MQ were collected and statistically treated.

The development and refinement of the questionnaire aimed to select the domains of primary importance for patient evaluation. The final questionnaire (MQ) consisted of five section: (1) general, (2) clinical examination, (3) radiological examination, (4) surface topography examination, (5) respondent's demographic data. The clinical examination comprised seven domains composed of 36 items (numbers of items in brackets): anthropometry (10 items), maturation (3), lower limbs discrepancy (4), trunk balance (3), sagittal plane (1), rib prominence (9), aesthetics (6). The radiological examination comprised twelve domains (79 items): patient positioning (12 items), cassette size (3), views (11), radiation protection (5), frontal plane parameters (6), rotation assessment (7), sagittal plane parameters (7), bone age (3), logistics (5), schedule (16), in-brace radiograph (2), follow-up (3). Surface topography examination comprised nine domains (72 items): hardware (5 items), patient positioning (5), views (3), logistics (13), anatomic landmarks (10), general and frontal plane parameters (17), sagittal plane parameters (8), transverse plane parameters (8) and pelvis (3). The final questionnaire is available as Additional File 2.

Questions were constructed to collect the participants' opinion on the usefulness of each particular parameter. The possible answers were as follows: 3 - always recommended; 2 - I use and recommend to use when it's needed; 1 - I don't use but it could be useful; 0 - never and I think it's not useful. In the Results section of this paper the score is presented as follows: Highest priority (3), Recommended (2), Acceptable (1) or Not recommended (0). Comments and complementary information could be freely provided at the end of each section. Each question was analyzed for agreement. The agreement for recommendation to use a particular parameter was calculated as the sum of the percentage of answers "always recommended (3)" and "I use and recommend to use when needed (2)" to the total number of participants who answered the question. SOSORT Scoliosis Evaluation Form was constructed with all items that received 75% of agreement or more and did not received more than 10% of "not recommended" answers while items that received 90% agreement or more were bolded as recommended for systematic use. SOSORT Scoliosis Evaluation Form is presented in Additional File 3.

## Results

Twenty-three filled-up MQ were collected during the Consensus Session. The respondents were 11 physicians, 10 physiotherapists and 1 orthotist; one person did not reveal the profession. Among the physicians there were six physiatrists specialized in conservative scoliosis treatment and five orthopaedic surgeons with experience in conservative management. Most respondents work in a team specialized in scoliosis management, composed most often of physician (physiatrist or orthopaedic surgeon), physiotherapist and orthotist. Half of the respondents see more than 10 scoliosis patients per week (table 1.).

**Table 1:** Characteristics of the respondents to the MQ (N = 23)

		number	%
Profession	Physician -- specialist in rehabilitation	6	26
	Physician -- orthopaedic surgeon	5	22
	Physiotherapist	10	43
	Orthotist	1	4
	Unknown	1	4

Institution	Public health system	6	26
	Private sector	12	52
	Both public and private	4	17
	Not answered	1	4

Scoliosis team (MD + PT + CPO)	Team work	15	65
	Alone	5	22
	Not answered	3	13

Number of patients with scoliosis evaluated per week	1 or less	1	4
	2 -- 5	3	13
	6 -- 10	5	22
	11 or more	12	52
	Not answered	2	8

MQ -- Meeting Questionnaire, MD -- physician, PT -- physiotherapist, CPO -- orthotists

The priority of methods of the assessment of the trunk morphology is reported in Table 2. The clinical examination has the highest priority, far before any other method. Radiographic assessment is recommended when needed, while always necessary for six participants.

**Table 2:** Priority of methods of assessment of the trunk morphology, N = 23

Method of assessment	High Prior	Rec	Acc	Not Rec	Not Ans
Clinical	19	2	1	0	1

Radiological	6	15	1	0	1

Surface topography	3	9	7	1	3

Photography	4	13	2	1	3

CT, MRI, US, Therm.	0	9	5	3	6

High Prior -highest priority, Rec - recommended, Acc -- acceptable, Not Rec - not recommended, Not Ans -- not answered, CT -- computer tomography, MRI -- magnetic resonance imaging, US -- ultrasounds, Therm. -- thermography;

Comments: (1) video filming is useful.

### Clinical examination

The data concerning clinical examination are presented in tables 3, 4, 5, 6 and 7 by percentage of indications to each answer option. The percentage of agreement concerning each parameter is calculated as the sum of the two columns (% of agreement = % highest priority + % recommended) to the total number of participants who answered the question (N).

**Table 3:** General anthropometry (% of indications)

Parameter	N	High Prior	Rec	Acc	Not Rec	% of Agreement
Weight	22	59.1	18.2	22.7	0.0	**77.3**

Height	23	82.6	13.0	4.3	0.0	**95.6**

Sitting height	23	26.1	34.8	30.4	8.7	**60.9**

Peak height velocity [13,14]	23	34.8	26.1	39.1	0.0	**60.9**

Arms span	23	21.7	21.7	34.8	21.7	**43.4**

Growth charts	23	26.1	30.4	34.8	8.7	**56.5**

Longitudinal graph	19	15.8	21.1	47.4	15.8	**36.9**

N -number of answers, High Prior -highest priority, Rec-recommended, Acc -- acceptable, Not Rec -not recommended

**Table 4:** Maturation data in girls (% of indications)

Parameter	N	High Prior	Rec	Acc	Not Rec	% of Agreement
Breast development (Tanner scale [11])	20	30.0	30.0	35.0	5.0	**60.0**

Pubic hair development (Tanner scale)	21	9.5	28.6	47.6	14.3	**38.1**

Menarche	21	95.2	0.0	4.8	0.0	**95.2**

N -number of answers, High Prior -highest priority, Rec - recommended, Acc -- acceptable, Not Rec -not recommended;

Comments: (1) stability of menses is a sign to be observed.

**Table 5:** Measurement of lower limbs discrepancy (% of indications)

Parameter	N	High Prior	Rec	Acc	Not Rec	% of Agreement
Lower limbs length assessment is necessary	19	78.8	15.8	5.3	0.0	**94.7**

ASIS level in standing position	23	65.2	26.1	8.7	0.0	**91.3**

PSIS level in standing position	22	63.6	9.1	22.7	4.5	**72.7**

ASIS -- malleolous in supine position	21	42.9	33.3	14.3	9.5	**76.2**

N -number of answers, High Prior -highest priority, Rec - recommended, Acc -- acceptable,

Not Rec -not recommended, ASIS -- anterior superior iliac spine. PSIS -- posterior superior iliac spine;

Comments: (1) assess the axis of lower limbs. (2) make the measures from the navel. (3) stretch hamstrings on the side of thoracic hump. (4) use sacral bone level in full bend. (5) measure distance trochanter-foot in side lying. (6) use shoe lift to level pelvis. (7) use iliac crests.

**Table 6:** Plumb line examination (% of indications)

Parameter	N	High Prior	Rec	Acc	Not Rec	% of Agreement
C7 plumb line	23	60.9	13.0	26.1	0.0	**73.9**

Axillary plumb line	21	23.8	4.8	66.7	4.8	**28.6**

Sagittal plumb line [3]	22	31.8	9.1	50.0	9.1	**40.9**

N -number of answers, High Prior -highest priority, Rec-recommended, Acc -- acceptable, Not Rec -not recommended;

Comment: (1) use inclinometer instead of plumb line for sagittal plane.

**Table 7:** Assessment of the transverse plane deformity (% of indications)

Parameter	N	High Prior	Rec	Acc	Not Rec	% of Agreement
Standing forward bending position [17]	23	82.6	8.7	4.3	4.3	**91.3**

Sitting forward bending position [18,19]	22	59.1	9.1	31.8	0.0	**68.2**

Prone position [36]	20	15.0	10.0	55.0	20.0	**25.0**

Scoliometer ATR measure main curve	22	95.0	0.0	4.5	0.0	**95.0**

Scoliometer ATR measure upper compensatory curve	21	85.7	0.0	14.3	0.0	**85.7**

Scoliometer ATR measure lower compensatory curve	22	90.9	0.0	9.1	0.0	**90.9**

Rib prominence measured in cm with a ruler	22	40.9	13.6	40.9	4.5	**54.5**

N -number of answers, High Prior -highest priority, Rec-recommended, Acc-- acceptable,

Not Rec -not recommended, ATR -- angle of trunk rotation (synonym ATI -- angle of trunk inclination);

Comment: (1) measure sacral bone rotation.

47.8% of respondents systematically note information about aesthetics, further 21.7% recommend it, totally 69.5% of agreement for recommendation. 43.5% only had a systematic way of noting aesthetics. Aesthetic Index and TRACE index [8] were presented during the Meeting and the respondents were questioned whether they knew the indices or found them useful. 56.5% found the indices useful, while 8.7% declared that use of photographs can replace it, further 8.7% did not consider it important.

### Radiological examination

The data are presented in tables 8, 9, 10, 11, 12, 13, 14, 15 and 16.

**Table 8:** Positioning of the patient (% of indications)

Parameter	N	High Prior	Rec	Acc	Not Rec	% of Agreement
Standing	23	95.7	4.3	0.0	0.0	**100**

Sitting	12	0.0	8.3	58.3	33.3	**8.3**

Supine	12	0.0	25.0	41.7	33.3	**25.0**

Prone	12	0.0	0.0	58.3	41.7	**0.0**

Spontaneous	19	78.9	10.5	10.5	0.0	**89.4**

Corrected	13	38.5	15.4	30.8	15.4	**53.9**

N -number of answers, High Prior -highest priority, Rec-recommended, Acc -- acceptable, Not Rec -not recommended;

Comment: (1) spontaneous should be neutral. every-day posture. not totally relaxed.

**Table 9:** Position of upper limbs for lateral radiography (% of indications)

Parameter	N	High Prior	Rec	Acc	Not Rec	% of Agreement
Along the trunk	18	44.4	0.0	11.1	44.4	**44.4**

Crossed on the chest	14	14.3	21.4	42.9	21.4	**35.7**

Reposed on a support	14	42.9	0.0	28.6	28.6	**42.9**

N -number of answers, High Prior -highest priority, Rec-recommended, Acc -- acceptable, Not Rec -not recommended;

Comments: (1) upper limbs bent. fingers on clavicles. (2) upper limbs bent 20 cm forward. (3) upper limbs in 45° anterior flexion.

**Table 10:** Cassette size and radiological views (% of indications)

Parameter	N	High Prior	Rec	Acc	Not Rec	% of Agreement
Long cassette	20	60.0	40.0	0.0	0.0	**100.0**

Standard cassette	15	20.0	46.7	13.3	20.0	**66.7**

Small cassette	9	0.0	0.0	22.2	77.8	**0.0**

Antero-posterior view	19	47.4	21.1	5.3	26.3	**68.5**

Postero-anterior view	16	62.5	31.3	6.3	0.0	**93.8**

Lateral view systematically	16	6.2	6.2	50.0	37.5	**12.5**

Lateral at start of treatment	20	75.0	20.0	5.0	0.0	**95.0**

Lateral at final visit	17	29.0	17.6	41.2	11.8	**46.6**

Oblique view of Stagnara [3]	16	0.0	12.5	50.0	37.0	**12.5**

Side bending	17	0.0	41.2	23.5	35.3	**41.2**

Supine traction	16	0.0	12.5	37.5	50.0	**12.5**

Axial for rib prominence	15	0.0	13.3	20.0	66.7	**13.3**

Wrist bone age	16	6.3	50.0	18.8	25.0	**56.3**

N -number of answers, High Prior -highest priority, Rec-recommended, Acc -- acceptable,

Not Rec -not recommended.

**Table 11:** Radiation protection (% of indications)

Parameter	N	High Prior	Rec	Acc	Not Rec	% of Agreement
Gonads	19	94.7	5.3	0.0	0.0	**100.0**

Breast	15	40.0	33.3	6.7	20.0	**73.3**

Thyroid	15	6.7	26.7	46.7	20.0	**33.4**

N -number of answers, High Prior -highest priority, Rec-recommended, Acc -- acceptable,

Not Rec -not recommended.

**Table 12:** Radiological parameters recommended for systematic use (% of indications)

Parameter	N	High Prior	Rec	Acc	Not Rec	% of Agreement
Cobb angle [37]	22	100.0	0.0	0.0	0.0	**100.0**

Fergusson angle [38]	15	0.0	6.7	46.7	46.7	**6.7**

C7 shift	20	35.0	25.0	35.0	5.0	**60.0**

Apical vertebra transposition	19	21.1	21.1	47.4	10.5	**42.2**

Nash and Moe rotation [39]	15	13.3	6.7	33.3	46.7	**20.0**

Drerup rotation [40]	12	0.0	0.0	66.7	33.3	**0.0**

Perdriolle rotation [41]	19	36.8	42.1	15.8	5.3	**78.9**

Raimondi rotation [42]	15	40.0	20.0	26.7	13.3	**60.0**

Mehta rib vertebra angle [43]	16	12.5	31.3	50.0	6.3	**43.8**

Segmental rib vertebra angle [44]	13	7.7	7.7	61.5	23.1	**15.4**

Th4-Th12 sagittal Cobb	22	50.0	40.9	9.1	0.0	**90.9**

L1-L5 sagittal Cobb	22	45.5	45.5	9.1	0.0	**91.0**

Lumbo-sacral L5-S1 angle	19	10.5	47.4	42.1	0.0	**57.9**

Double rib contour sign [45]	17	5.9	29.4	58.8	5.9	**35.3**

Sacral slope	18	16.7	27.8	55.6	0.0	**44.5**

Pelvic incidence [46]	18	22.2	27.8	50.0	0.0	**50.0**

Risser sign [23]	21	90.5	9.5	0.0	0.0	**100.0**

Triradiate cartilage closure	16	18.8	12.5	56.3	12.5	**31.3**

N -number of answers, High Prior -highest priority, Rec - recommended, Acc -- acceptable, Not Rec -not recommended;

Comments - additional parameters to be used systematically: (1) imbalance of transitional point. (2) transitional point to CSL. (3) sagittal segmental evaluation. (4) restricted kyphosis angle [48]. (5) wrist bone age. (6) elbow bone age.

**Table 13:** Where the X-ray should be made? (% of indications)

Parameter	N	High Prior	Rec	Acc	Not Rec	% of Agreement
At any X office of patient's choice	15	26.7	20.0	33.3	20.0	**46.7**

At indicated X-ray office only	19	57.9	26.3	10.5	5.3	**84.2**

N -number of answers, High Prior -highest priority, Rec - recommended, Acc -- acceptable,

Not Rec -not recommended

**Table 14:** Schedule for X-ray examination (% of indications)

Parameter	N	High Prior	Rec	Acc	Not Rec	% of Agreement
First visit						

Always	14	21.4	21.4	21.4	35.7	**42.8**

Only if clinically suspected	18	83.3	11.1	5.6	0.0	**94.4**

Intervals while management with observation						

3 months	14	7.1	21.4	35.7	36.7	**28.5**

6 months	17	29.4	47.1	5.9	17.6	**76.5**

12 months	16	62.5	25.0	6.3	6.3	**87.5**

Other	7	28.6	14.3	14.3	42.9	**42.9**

Intervals while management with physiotherapy						

3 months	14	7.1	21.4	21.4	50.0	**28.5**

6 months	17	23.5	29.4	17.6	29.4	**52.9**

12 months	16	50.0	31.3	6.3	12.5	**81.3**

Other	7	14.3	14.3	28.6	42.9	**28.6**

Intervals while management with brace						

3 months	15	20.0	33.3	6.7	40.0	**53.3**

6 months	16	31.3	43.8	0.0	25.0	**75.1**

12 months	13	61.5	23.1	7.7	7.7	**84.6**

Other	4	25.0	25.0	25.0	25.0	**50.0**

Comments: (1) in puberty 6 months. (2) depends on suspected growth rate. (3) depends on child age. (4) 6 months at 11-13 years. 12 months at 13-15 years. (5) when clinical progression. (6) every 4 months.

N -number of answers, High Prior -highest priority, Rec-recommended, Acc -- acceptable,

Not Rec -not recommended.

**Table 15:** Type of radiograph performed during brace treatment (% of indications)

Parameter	N	High Prior	Rec	Acc	Not Rec	% of Agreement
In brace	19	68.4	15.8	15.8	0.0	**84.2**

Out of brace	17	58.8	29.4	11.8	0.0	**88.2**

N -number of answers, High Prior -highest priority, Rec - recommended, Acc -- acceptable,

Not Rec -not recommended.

**Table 16:** Follow-up considered the outcome of brace treatment (% of indications)

Parameter	N	High Prior	Rec	Acc	Not Rec	% of Agreement
1 year after completion of treatment	15	60.0	33.3	6.7	0.0	**93.3**

2 years after completion of treatment	14	85.7	0.0	14.3	0.0	**85.7**

N -number of answers, High Prior -highest priority, Rec - recommended, Acc -- acceptable,

Not Rec -not recommended;

Comment: (1) 6 months.

The respondents were asked who should perform the radiological measurements, having: (1) the treating person, (2) the radiologist or (3) other person as answer option. All but two physicians (N = 9) highly recommended the measurements done by the treating physician (expert) and they excluded the radiologist. The two remaining accorded the measures to be done by the radiologist. The non-physician responders divided in opinions equally between the radiologist and the treating physician but never indicated themselves.

### Surface topography examination

The number of participants to this section was eleven, six were physicians and five were physiotherapists. The remaining respondents declared to be not enough experienced with surface topography to answer the questionnaire. The equipment used was basically raster stereography method (Formetric, Orten, CQ, other), Auscan, Goals and classical Moire. No respondent revealed being used the Quantec system or ISIS technique. The data is presented in tables 17, 18, 19 and 20. The questions concerning the surface parameters recommended for systematic use were answered by eight responders only: four physicians and four physiotherapists (table 20).

**Table 17:** Logistics of surface topography examination (% of indications)

Parameter	N	High Prior	Rec	Acc	Not Rec	% of Agreement
Availability of the hardware						

At the practise	19	68.4	15.8	15.8	0.0	**84.2**

Out of practise but easily accessible	17	58.8	29.4	11.8	0.0	**88.2**

Who performs surface topography examination						

Physician	7	14.3	0.0	14.3	71.4	**14.3**

Physiotherapist	8	62.5	12.5	12.5	12.5	**75.0**

Technician	7	14.3	0.0	14.3	71.4	**14.3**

Nurse	6	16.7	0.0	16.7	66.7	**16.7**

Who treats surface images and performs measurements						

Physician	8	25.0	12.5	12.5	50.0	**37.5**

Physiotherapist	8	62.5	12.5	12.5	12.5	**75.0**

Technician	6	0.0	0.0	16.7	83.3	**0.0**

Nurse	6	0.0	0.0	16.7	83.3	**0.0**

Final interpretation of surface topography exam is basically on						

Images created	8	75.0	12.5	0.0	12.5	**87.5**

Values of parameters	10	90.0	10.0	0.0	0.0	**100.0**

N -number of answers, High Prior -highest priority, Rec - recommended, Acc -- acceptable,

Not Rec -not recommended.

**Table 18:** Positioning of the patient for surface topography (% of indications)

Parameter	N	High Prior	Rec	Acc	Not Rec	% of Agreement
Position						

Standing upright	11	100.0	0.0	0.0	0.0	**100.0**

Standing forward bent	7	28.6	14.3	28.6	28.6	**42.9**

Sitting upright	7	14.3	14.3	42.9	28.6	**28.6**

Sitting forward bent	8	12.5	25.0	37.5	25.0	**37.5**

View						

Back	10	100.0	0.0	0.0	0.0	**100.0**

Front	7	14.3	14.3	28.6	42.9	**28.3**

N -number of answers, High Prior -highest priority, Rec-recommended, Acc -- acceptable,

Not Rec -not recommended;

Comments: (1) movements performed: autocorrection. side bending. (2) left and right bending.

(3) three-dimensional view.

**Table 19:** Anatomic surface landmarks to be taken into consideration systematically (% of indications).

Parameter	N	High Prior	Rec	Acc	Not Rec	% of Agreement
Spinous processes	10	90.0	10.0	0.0	0.0	**100.0**

Posterior iliac spines	10	90.0	10.0	0.0	0.0	**100.0**

Rib prominence	8	87.5	0.0	0.0	12.5	**87.5**

Occiput	7	42.9	0.0	14.3	42.9	**42.9**

Neck	7	71.4	14.3	0.0	14.3	**85.7**

Shoulders	10	80.0	20.0	0.0	0.0	**100.0**

Scapulae	9	77.8	11.1	0.0	11.1	**88.9**

Waist	8	62.5	12.5	12.5	12.5	**75.0**

Coccyx	8	62.5	12.5	12.5	12.5	**75.0**

N -number of answers, High Prior -highest priority, Rec-recommended, Acc -- acceptable,

Not Rec -not recommended

Comment: (1) heels systematically.

**Table 20:** Surface parameters recommended for systematic use (% of indications)

Parameter	N	High Prior	Rec	Acc	Not Rec	% of Agreement
General						

Spine length (curve line)	9	88.9	11.1	0.0	0.0	**100.0**

Spine height: C7-S1 distance	9	55.6	11.1	11.1	22.2	**66.7**

Measures of main curve only	8	37.5	0.0	12.5	50.0	**37.5**

Measures of main and secondary curves	8	100.0	0.0	0.0	0.0	**100.0**

Body axis definition						

Analogous to radiological VCSL	7	100.0	0.0	0.0	0.0	**100.0**

C7-S1 line	7	85.7	0.0	14.3	0.0	**85.7**

Frontal plane analysis						

C7 shift	8	62.5	0.0	12.5	25.0	**62.5**

Curve angle	8	75.0	0.0	0.0	25.0	**75.0**

Apex distance from body axis	7	71.4	0.0	0.0	28.6	**71.4**

Frontal plane body asymmetry						

Shoulders	9	66.7	0.0	0.0	33.3	**66.7**

Scapulae	9	66.7	0.0	0.0	33.3	**66.7**

Waist	8	50.0	0.0	12.5	37.5	**50.0**

Special indices						

POTSI index [48,49]	7	28.6	0.0	0.0	71.4	**28.6**

Weiss index	7	14.3	14.3	14.3	57.1	**28.6**

Sagittal plane analysis						

Relation of C7 to S1	8	87.5	12.5	0.0	0.0	**100.0**

Cervical lordosis	9	77.8	22.2	0.0	0.0	**100.0**

Thoracic kyphosis	9	100.0	0.0	0.0	0.0	**100.0**

Lumbar lordosis	9	100.0	0.0	0.0	0.0	**100.0**

Segmental analysis	6	50.0	0.0	50.0	0.0	**50.0**

Limits of kyphosis and lordosis indicated						

Automatically by software	7	85.7	14.3	0.0	0.0	**100.0**

Manually by examinator	6	33.3	33.3	0.0	33.3	**66.6**

Transverse plane analysis						

Trunk rotation main curve	8	100.0	0.0	0.0	0.0	**100.0**

Trunk rotation compensatory curves	8	100.0	0.0	0.0	0.0	**100.0**

Hump Sum [49,50]	7	42.9	42.9	14.3	0.0	**85.8**

DAPI index [51]	5	0.0	0.0	80.0	20.0	**0.0**

Levels to measure surface rotation indicated						

Automatically by software	6	83.3	0.0	16.7	0.0	**83.3**

Manually by examinator	6	50.0	16.7	0.0	33.3	**66.7**

Pelvis						

PSIS height	7	100.0	0.0	0.0	0.0	**100.0**

PSIS depth (distance from the camera)	7	71.4	0.0	0.0	28.6	**71.4**

Manual correction of patient position to achieve pelvis level at PSIS	7	42.9	14.3	14.3	28.6	**57.2**

N -number of answers, High Prior -highest priority, Rec-recommended, Acc -- acceptable,

Not Rec -not recommended, VCSL -- vertical central sacral line. PSIS -- posterior superior iliac spines.

## Discussion

The general opinion (table 2) of the respondents was that clinical examination remains essential in evaluation of the patient with spinal deformity. Radiography tends to be used "when needed". There was agreement on the necessity of documentation of the surface shape of the trunk, the classical photography still tends to be more widely accepted than surface topography. It was probably due to the fact that as many as 13 responders refused fulfilling the surface topography section because of insufficient experience. The use of CT, MRI, US or thermography had never the highest priority in the assessment of trunk morphology.

### Maturation (table 4)

It is recognized that progression of structural scoliotic curvature takes place essentially during the periods of rapid spinal growth [9]. Generally, in adolescent girls, this period corresponds to two pre-menarchial and one post-menarchial year [10]. Thus, the onset of puberty should be observed and documented. The first pubic hair and first breast development are clinical signs easy to be disclosed. They are graded P2 and S2 in the Tanner scale of sexual maturation [11]. Duval-Beaupere considered this moment the onset of puberty (P point at the schema of Duval-Beaupere), the turning point in scoliosis progression [12]. Detection of point P is of extremely importance in conservative management of idiopathic scoliosis, more than in case of surgically oriented approach, because it is essential to detect the curve at risk of progression before progression takes place. Unfortunately, in the current social scenario with people being very sensitive to social issues and the actual problem of pedophilia, an innocent clinical inspection of sexual maturation risk to be misconstrued. There is no doubt it can be done with respect to sensitivity and shyness of a young girl, if not, it can be replaced by investigating the mother but never missed. It is to notice that Tanner stages do not need to be checked after menarche occurs.

To calculate the **peak height velocity **it is necessary to dispose of the patient's height noted at regular intervals of 6 months, at least 3 times. The value of 9 cm/year (occurring in average 12 years chronological age in girls) was reported to be a good predictor for curve progression, better than Risser sign or menarche [13]. For Sanders et al. [14] the peak height velocity could predict the crankshaft phenomenon.

### Lower limbs discrepancy (table 5)

Assessment of the length of the lower limbs was recommended by the participants, essentially in standing position with anterior superior iliac spines (ASIS) as reference points. It was reported that the position of ASIS is sensitive to intra-pelvic distortion due to nutation/counter-nutation movements of the hemipelvis [15]. The level of femoral heads observed on a long cassette standing radiograph remains precise way of measuring the actual lower limbs discrepancy.

### Plumb line exam (table 6)

The examination with the plumb line has entered the classics of scoliosis measurements [3]. It appears less popular today, however it was still recommended in case of the assessment of C7 lateral shift.

### Transverse plane of deformity (table 7)

The recommendation was made to measure the angle of trunk rotation (ATR, synonym ATI - angle of trunk inclination, [16]) with a scoliometer within the main curvature as well as within both the upper and the lower compensatory curvatures. The position is standing forward bending (classical Adams test [17]); the sitting forward bending position [18,19] was also recommended. The sitting position claims to eliminate pelvic obliquity due to lower limb discrepancy.

Examination of trunk asymmetries with a scoliometer performed in sitting position seems to be useful when detecting small deformities, for example for the purpose of scoliosis screening. Grivas reported higher percentage of symmetric children when examined in sitting versus standing position [18]. The hypothesis is that the specificity of the examination increases in sitting position because discrete asymmetries of the back due to lower limbs discrepancy, hip joints pathology or pelvic distortion are eliminated.

Assessment of the height of rib prominence in Adams test can be practiced without scoliometer, with the use of a simple ruler. The measurement error was estimated +/- 2 mm [20]. For Duval-Beaupere the rib prominence height of 11 mm or more combined with supine Cobb angle of 17 degrees or more indicated progressive curves [20].

### Sagittal plane

The Meeting Questionnaire did not approach deeply the issue of clinical assessment of sagittal balance (one question only). The sagittal plane assessment was the main topic of the 2009 SOSORT Annual Meeting in Lyon [21].

#### Radiological examination

There is a clear recommendation for standing position to make spinal radiographs. The posture should be neutral, spontaneous which means not actively corrected on one hand and not totally relaxed on the other hand (table 8). There was no agreement for the position of the upper limbs during lateral radiography to avoid its superposition over the spine (table 9). Long cassette radiographs are highly recommended, small size X-rays rejected (table 10). Postero-anterior view, or eventually antero-posterior view are for systematic use. Lateral view is not considered regular, but ordered in selected moments, for instance when the brace treatment begins. Radiation protection should systematically consider the gonads (table 11). Axially loaded MRI was proposed to avoid radiation when measuring Cobb angle [22]. 84.2% of respondents recommended the patient to be guided to make the X-ray in a dedicated office (table 13). Probably, the local circumstances decide whether the X-rays are considered reliable regardless the office they are made.

### Parameters for systematic use

Among the parameters for systematic use, the Cobb angle and the Risser sign got 100% of recommendations, followed by sagittal thoracic and lumbar Cobb angles (over 90%). Perdriolle method revealed the most recommended for measuring the vertebral axial rotation (78.9%). It is understandable that other radiological measures got less indications, as this question was constructed to reveal the parameters considered in a systematic way in clinical practise. The Questionnaire did not precise whether the original [23] or the so-called French [24] version is recommended to be used. Both versions differ each from other in description of grades 3 and 4; they can be improved in accuracy with lateral spinal radiography [25].

#### Surface topography

Petit et al. stated: "Because surgeons are so familiar with Cobb angle measurements on radiograph, the introduction of new surface shape measures whose meaning may not be readily apparent to clinicians has been difficult" [26]. The section concerning surface topography examination revealed to be the most difficult to the participants: 13 of 23 did not filled-up this part of the questionnaire. The explanation appeared insufficient experience in surface topography.

In last three decades we observed a dynamic development of the equipment and software for surface measurements. The International Research Society on Spinal Deformities (IRSSD) started in the 80 s of last century as working group for Moire Topography, Surface Topography and 3 Dimensional Spinal Deformity. Current technical capabilities offer reliable and precise surface measurements. Unfortunately, clinicians apparently did not take advantage of the technique. The reason does not seem to be the lack of consensus. Also, in this study, a good consensus for surface measurements was achieved, as good as for clinical and radiological exams.

Searching for relationship between radiological Cobb angle and surface parameters with making presumption that the higher correlation with Cobb angle, the better the surface technique may be one of the reason that introduced the surface topography in a blind alley. In fact, Cobb angle is nothing more than a shadow of two limit vertebrae. It is not clear what would be the rationale to expect that so constructed angle should highly correlate with any of the surface describing parameters. James presented photos of four girls with 70° curvature each to demonstrate the variations in external shape of the back [27]. Thulbourne and Gillespie indicated that the rib hump does not obligatory follow the Cobb angle [28]. Asher and Manna found discrepancy between surgical reduction of the Cobb angle and reduction of trunk asymmetry [29]. Furthermore, Hackenberg reported the same phenomenon concerning operative correction of radiological vertebral rotation versus the surface rotation measured with raster-stereography [30]. As stated by Goldberg et al. the surface and the x-rays simply "are not measuring the same aspect of the deformity" [31]. We share the opinion of Dubousset that in idiopathic scoliosis "the soft tissues are more important than the bones" [Dubousset J: Personal communication, unpublished].

Gold standard parameters for surface topography wait to be named. It is also a challenge for producers of surface topography equipment to unify the parameters in order to let the users speak the same language. In our opinion, surface topography seems still have a chance to work in service of patients if additional effort in order to standardize the examination is done.

## Conclusion

This paper is the 6^th ^SOSORT Consensus Paper. Previously, the role of physical exercises [32], the aims of treatment [5], the biomechanics of brace action [33], the school screening [34], and the quality of brace treatment [35] were debated.

According to the results obtained the authors prepared "Proposal for SOSORT Scoliosis Evaluation Form" (Apendix 3). It is offered as proposal to professionals taking care of the patients with idiopathic scoliosis. It is supposed to serve as a guideline for clinicians and a tool for patients to check the course of the disease. It was based on the Consensus, completed according to the authors' experience and may be further developed with new parameters to perform high quality clinical research studies.

This study addresses the issue of scoliosis evaluation and emphasizes the need for correct methodological assessment of the shape of the spine and the whole body. Hence, it should be stressed that methodology limited to Cobb angle analysis seems to be particularly misleading. Moreover, the authors would like to underline that describing such a complex condition with exclusively morphology-related parameters is superficial. Morphology alone cannot substitute the assessment of the impaired function as well as of the quality of life.

## Competing interests

The authors declare that they have no competing interests.

## Authors' contributions

T.K. - study design, collecting data, data analysis and interpretation, manuscript drafting S.N., T.B.G., M.R., T.M., J.D., F.Z. - data analysis and interpretation, manuscript revision All authors read and approved the final manuscript.

## Supplementary Material

Additional file 1 **Pre-Meeting Questionnaire**. the first version of the consensus questionnaire. Click here for file

Additional file 2 **Meeting Questionnaire**. the final version of the consensus questionnaire. Click here for file

Additional file 3 **Proposal for SOSORT Scoliosis Evaluation Form**. evaluation form for scoliosis recommended by the authors. Click here for file
