# Supplementary material for: Methodology of evaluation of morphology of the spine and the trunk in idiopathic scoliosis and other spinal deformities - 6th SOSORT consensus paper
Source: Scoliosis. 2009 Nov 26;4:26. doi: 10.1186/1748-7161-4-26 (PMC2794256; doi:10.1186/1748-7161-4-26)
Supplement: Additional file 1 — Pre-Meeting Questionnaire. the first version of the consensus questionnaire. [file 1748-7161-4-26-S1.DOC]

SOSORT Questionnaire for preparing the Consensus Session:

„Measurements”

Criteria for the assessment of the outcome

of non-operative scoliosis management

*Please use Y (Yes) or N (No) by deleting the inappropriate one*

*or marking the proper one with another colour*

*and provide complementary information in blank spaces*

Introduction

The assessment of the outcome of scoliosis therapy considers:

1. Morphology

2. Function

3. Quality of Life

The **MORPHOLOGY** only is the subject of this study.

The **Function** and the **Quality of Life** merit separate Consensus Sessions.

The assessment of the morphology of a scoliotic subject considers:

1. Clinical examination including scoliometer measurements

2. Radiological examination

3. Surface topography examination

4. Rare: ultrasounds ? (Burwell) **Y/N**, thermography ? **Y/N**

CT scans **Y/N**, Magnetic Resonance **Y/N**

5. Classical photography **Y/N**

6. Others – please indicate : …

Ad.1. Clinical examination

The visual assessment of the body is continuously performed by the patient, the family and the treating team. The body proportions, the relationship of various parts of the body and the posture are analyzed. Moreover the appraisal of the patient morphology can be made both for the static and dynamic conditions (gait).

Traditionally the clinical examination includes the assessment of asymmetries of the shoulders, scapulae, flanks, hips, the plumb line exam, trunk imbalance, disturbances in sagittal curvatures and rotational phenomena (rib hum, lumbar prominence).

The findings of such an exam are usually noted in a qualitative manner, for example:

“Right shoulder in elevation and anteposition comparing to the left shoulder”

“Thoracic kyphosis markedly reduced”

“Protruding left hip” “Protruding inferior angle of the right scapula” etc.

The skills concerning observation of trunk asymmetries are important in detecting mild scoliosis. The clinical examination should be carefully learned by medical students. However it may be relatively useless in providing data suitable for scientific analysis of the result of scoliosis therapy because of its qualitative nature.

*The following clinical parameters are proposed to be discussed:*

*(please indicate your opinion with* ***Y-N*** *and provide supplementary information in blank spaces)*

**General**:

- age of birth Y-N
- weight (kg) Y-N
- height (cm) Y-N
- sitting height (cm) Y-N
- arms span Y-N
- comparison with statistic percentiles (growth charts) Y-N
- drawing a graph of longitudinally collected data Y-N
- skin and eye colour Y-N
- others …

**Maturation**:

- Tanner scale of maturation Y-N

Breast: Stage 1 through 5 Y-N

Pubic hair: Stage 1 trough 5 Y-N

- others…

**Lower limb discrepancy**:

Assessment in standing position:

- anterior superior iliac spines level Y-N
- posterior superior iliac spines level Y-N
- others…

Assessment in supine position:

- distance from anterior superior iliac spine to the tip of the medial malleolous measured in cm Y-N

- others…

**Trunk balance**:

- deviation of the plumb line from C7 to the side

(measured in centimeters) Y-N

- left or right axillary’s plumb line measured

to great trochanter (cm) Y-N

- others….

**Sagittal** distance in the deepest part of cervical or lumbar lordosis

(in cm) Y-N

**Rib hump** or lumbar prominence measured in forward bending,

with one horizontal device and one vertical ruler

(in millimeters), Y - N

**Trunk rotation measured with scoliometer (ATR or ATI**), (degrees)

- main curve Y-N
- lower compensatory curve Y-N
- upper compensatory curve Y-N

others…

**Position to examine spine rotation (ATR, ATI)**

1. -Standing FBT *
2. -Sitting FBT
3. -Prone
4. -Combination of (please list): ……………………………………………………………

*The standing forward bending test (FBT) traditionally refers to the Adams Forward Bending Test; however, recently some additional body positions have been utilized; i.e., the sitting or prone positions. For this reason, we are herein substituting the terms Standing FBT, Sitting FBT or Prone Position for the Adams Forward Bending Test.

Additional comments:

_________________________________________________

There exist two other issues in clinical examination:

1. **Clinical tests of the flexibility** of the curve

(side bending, traction, prone exam)

2. **Detecting non-idiopathic** nature of the curve

(skin spots, joint hyperlaxity, abdominal reflexes etc.).

It is proposed not to integrate these two problems in this consensus session. The flexibility might be incorporated in a future FUNCTION Consensus Session as a part of **spine mobility**. The second point by pre assumption should demonstrate the idiopathic etiology.

Y-N Comments: _______________________________________

_______________________________________________________

Ad. 2. Radiological examination

*Please mark your answer with a X, or mark Yes or No,*

*or give additional comments*

1) position of the patient for the X-ray

Standing

Sitting

Supine

Prone

Comments:

2) posture

Relaxed, spontaneous

Corrected

(corrected how ?)

Comments:

2a) position of upper limbs while the child is radiographed in standing position for the lateral view:

Along the trunk

Crossed on the chest

Reposed at special support

Other….

3) cassette size

Long cassette (80-90 cm long)

Standard cassette (35-40-43 cm long)

Small sizes (which?)

Comments:

4) view

A-P always

Lateral: always

Often (how often?)

At the beginning of treatment

At the final visit

At regular interval (once a year? , more rarely?)

Comments:

5) special views:

- oblique “plan d’election” de Stagnara Y-N
- side bending Y-N
- supine traction Y-N
- axial for rib hump Y-N
- left hand for bone age (Greulich-Pyle) Y-N
- others (which?) …

Comments:

6) radiation protection

Systematic or not: (Y-N)

Gonads

Breast

Thyroid

Others (which?)

The first radiograph usually is taken without any radiation protection thus any anomaly could be exposed and described. Y-N

Comments:

7) Parameters for systematic use (your recommendation).

Cobb angle (in degrees) Y-N

Fergusson angle (in degrees) Y-N

Others:

C7 shift Y-N

Apical vertebra transposition Y-N

Others:

Axial rotation of vertebra:

Nash and Moe grades Y-N

Perdriolle Y-N

Drerup Y-N

Mehta rib-vertebra angle (RVA apical) Y-N

Segmental RVAs Y-N

Others ……

Kyphosis angle Th4(5)-Th12 Y-N

Lordosis angle L1-L5 Y-N

Lumbo-sacral angle (L5-S1) Y-N

Double rib contour sign Y-N

Others for sagittal plane ….

Sacral slope Y-N

Pelvic incidence Y-N

Risser sign Y-N

Triradiate cartilage stage (open - closed)

Others for bone age… Y-N

Comments:

8) Radiological measurements are made by:

Myself (the treating person)

Radiologist

Other

9) Schedule for X-ray exam

First visit:

Always Y-N

Only if clinically suspected Y-N

Place for the X-ray examination:

Any X-ray office of patient’s choice Y-N

Indicated X-ray office only Y-N

Interval between subsequent X-rays during observation:

3 months Y-N

6 months Y-N

12 months Y-N

Other Y-N

Interval between subsequent X-rays during management with physiotherapy:

3 months Y-N

6 months Y-N

12 months Y-N

Other Y-N

Interval between subsequent X-rays during brace treatment:

3 months Y-N

6 months Y-N

12 months Y-N

Other Y-N

Type of radiograph during brace treatment:

In brace Y-N

Out of brace Y-N

Comments:

_________________________________________________

Follow-up for the final X-ray for the outcome of treatment:

1 year after completion of treatment Y-N

2 years after Y-N

Other

Other comments concerning radiographic exam:

Ad. 3. Surface topography measurements

1) Hardware

Formetric

Another raster stereographic

ISIS

Quantec

Moire

Surphaser

Others

2) Position

Standing

Sitting

Other

3) View

Back

Front

Other

4) Surface topography hardware available

At the place of my practice

At proximity, easily accessible

Not available

5) Surface topography examination

(patient’s positioning, skin markers etc.) is made by:

Myself (the treating person)

Physiotherapist

Nurse

Technician

Other

6) Surface topography measurements

(choosing points, drawing lines at the computer screen etc. )

are made by:

Myself (the treating person)

Physiotherapist

Nurse

Technician

Other

7) When using surface topography, you make interpretation

of the exam basically on:

Image created by the software Y-N

Values of parameters calculated by the software Y-N

8) Anatomic landmarks which should be systematically

taken into consideration: (*mark with X*)

Spinous processes

Posterior iliac spines

Rib prominence (rib hump)

Occiput

Neck

Shoulders

Scapulae

Waist

coccyx

others

9) Surface topography parameters

(your recommendation for systematic use):

**General:**

Spine length (curve line) Y-N

Spine height (C7-S1 straight distance) Y-N

Body axis definition:

Analogous to radiological central sacral line Y-N

Other

**Frontal plane analysis:**

C7 shift Y-N

Curve angle Y-N

Apex distance from the body axis Y-N

Other

Measurements for main curve only Y-N

Measurements for main and secondary curves Y-N

Body asymmetry in the frontal plane:

Shoulders Y-N

Scapulae Y-N

Waist Y-N

Special indices:

POTSI Y-N

others

**Sagittal plane analysis:**

C7 relation to S1 Y-N

Cervical lordosis Y-N

Thoracic kyphosis Y-N

Lumbar lordosis Y-N

The limits (vertebral levels) for measuring thoracic kyphosis

and lumbar lordosis, are they :

Automatically indicated by the software Y-N

Manually indicated Y-N

Segmental analysis of the profile available Y-N

Segmental analysis of the profile desirable Y-N

Other

**Transverse plane analysis:**

Trunk rotation in main curve Y-N

Trunk rotation in compensatory curves Y-N

Special indices: HUMP SUM Y-N

Others

The level to measure trunk rotation is indicated:

Automatically by the software Y-N

Manually by the person analyzing data Y-N

other

**Pelvis:**

Posterior superior iliac spines height Y-N

Posterior superior iliac spines depth

(difference in the distance from the camera) Y-N

Pelvis position corrected before examination

to achieve iliac spines level at height and depth Y-N

Other comments for surface topography:

*Thank you for providing your experience !*

The answers to the questionnaire will be reported as pooled data only, i.e., the individual respondent's answers will be kept confidential.

**Questionnaire respondent’s demographics**

Name: ……………………………………………………

Surname: …………………………………………

Specialty: ………………………………………

I am working under the:

1. o National (public) health system
2. o Private sector

Institution’s name: …………………………...

Address: ………………………………………………

Country/State: ……………………………….

e-mail: ………………………………………………..

Fax: ……………………………… Tel: ………………………………………………..

Please send the questionnaire to:

kotwicki@ump.edu.pl
