# Supplementary material for: Methodology of evaluation of morphology of the spine and the trunk in idiopathic scoliosis and other spinal deformities - 6th SOSORT consensus paper
Source: Scoliosis. 2009 Nov 26;4:26. doi: 10.1186/1748-7161-4-26 (PMC2794256; doi:10.1186/1748-7161-4-26)
Supplement: Additional file 2 — Meeting Questionnaire. the final version of the consensus questionnaire. [file 1748-7161-4-26-S2.DOC]

SOSORT Questionnaire for the Consensus Session:

„Measurements”

Criteria for the assessment of the outcome

of non-operative scoliosis management

-----------------------------------------------------------------------------------------------------------------

Please use the following answers:

0 – Never, and I think it’s not useful

1 – I don’t use, but it could be useful

2 – I use and recommend to use when it’s needed

3 – Always recommended

You may provide complementary information in blank spaces

-----------------------------------------------------------------------------------------------------------------

Introduction

The assessment of the outcome of scoliosis therapy considers:

1. Morphology

2. Function

3. Quality of Life

The **MORPHOLOGY** only is the subject of this study.

The **Function** and the **Quality of Life** merit separate Consensus Sessions.

The assessment of the morphology of a scoliotic subject should consider:

1. Clinical examination including scoliometer measurements 0-1-2-3

2. Radiological examination 0-1-2-3

3. Surface topography examination 0-1-2-3

4. Classical photography 0-1-2-3

5. Rare: ultrasounds, thermography, CT scans, MRI 0-1-2-3

6. Others – … 0-1-2-3

Part A. Clinical examination

The visual assessment of the body is continuously performed by the patient, the family and the treating team. The body proportions, the relationship of various parts of the body and the posture are analyzed. Moreover the appraisal of the patient morphology can be made both for the static and dynamic conditions (gait).

Traditionally the clinical examination includes the assessment of asymmetries of the shoulders, scapulae, flanks, hips, the plumb line exam, trunk imbalance, disturbances in sagittal curvatures and rotational phenomena (rib hum, lumbar prominence).

The findings of such an exam are usually noted in a qualitative manner, for example:

“Right shoulder in elevation and anteposition comparing to the left shoulder” “Thoracic kyphosis markedly reduced” “Protruding left hip” “Protruding inferior angle of the right scapula” etc.

The skills concerning observation of trunk asymmetries are important in detecting mild scoliosis. The clinical examination should be carefully learned by medical students. However it may be relatively useless in providing data suitable for scientific analysis of the result of scoliosis therapy because of its qualitative nature.

**A1. General parameters**:

A1.1 age of birth 0-1-2-3

A1.2 weight (kg) 0-1-2-3

A1.3 height (cm) 0-1-2-3

A1.4 sitting height (cm) 0-1-2-3

A1.5 peak height velocity 0-1-2-3

A1.6 arms span 0-1-2-3

A1.7 comparison with statistic percentiles (growth charts) 0-1-2-3

A1.8 drawing a graph of longitudinally collected data 0-1-2-3

A1.9 skin and eye color 0-1-2-3

A1.10 others …

**A2. Maturation**:

A2.1 Tanner scale of maturation for development of:

Breast: Stage 1 through 5 0-1-2-3

Pubic hair: Stage 1 trough 5 0-1-2-3

A2.2 Age of menarche 0-1-2-3

A2.3 others…

**A3. Lower limb discrepancy**: 0-1-2-3

Assessment in standing position:

A3.1 anterior superior iliac spines level 0-1-2-3

A3.2 posterior superior iliac spines level 0-1-2-3

A3.3 others…

Assessment in supine position: distance from anterior iliac spine to the tip of the medial malleolous (cm) 0-1-2-3

A3.4 others…

**A4. Trunk balance**:

A4.1 deviation of the plumb line from C7 to the side (cm) 0-1-2-3

A4.2 left or right axillary’s plumb line to great trochanter (cm) 0-1-2-3

A4.3 others….

**A5. Sagittal** distance from plumb line to the deepest part of cervical or lumbar lordosis

(cm) 0-1-2-3

**A6. Rib prominence** or lumbar prominence measured in forward bending,

with one horizontal device and one vertical ruler (cm) 0-1-2-3

**A7. Trunk rotation measured with scoliometer (ATR or ATI***, degrees)

A7.1 main curve 0-1-2-3

A7.2 lower compensatory curve 0-1-2-3

A7.3 upper compensatory curve 0-1-2-3

A7.4 others…

* ATR Angle of Trunk Rotation, ATI Angle of Trunk Inclination

**A8. Position to examine spine rotation with scoliometer (ATR, ATI)**

1. A8.1 Standing Forward Bending (classical Adams)* 0-1-2-3
2. A8.2 Sitting Forward Bending 0-1-2-3
3. A8.3 Prone 0-1-2-3

A8.4 Other (please describe): ……………………………………………………………

*The standing forward bending test (FBT) traditionally refers to the Adams Forward Bending Test; however, recently additional positions have been utilized; i.e., the sitting or prone positions. For this reason, we are herein substituting the terms Standing FBT, Sitting FBT or Prone Position for the Adams Forward Bending Test.

**A9. Aesthetic clinical evaluation**

A9.1 Do you write anything about aesthetics on your clinical form ? 0-1-2-3

A9.2 Do you have any systematic way of writing it ? 0-1-2-3

A9.3 Do you think that the Aesthetic Index and the TRACE instruments presented at this meeting could be useful:

A9.4 yes, and I will try them

A9.5 yes, but I will not try them (reason: ..............................................)

A9.6 no

Other comments on clinical examination issues:

Part B. Radiological examination

-----------------------------------------------------------------------------------------------------------------

Please use the following answers:

0 – Never, and I think it’s not useful

1 – I don’t use, but it could be useful

2 – I use and recommend to use when it’s needed

3 – Always recommended

You may provide complementary information in blank spaces

-----------------------------------------------------------------------------------------------------------------

**B1. position of the patient for the X-ray** B1.1 Standing 0-1-2-3

B1.2 Sitting 0-1-2-3

B1.3 Supine 0-1-2-3

B1.4 Prone 0-1-2-3

B1.5 Other 0-1-2-3

**B2. posture** B2.1 Relaxed, spontaneous 0-1-2-3

B2.2 Corrected 0-1-2-3

B2.3 Comment…

B3. position of upper limbs while the child is radiographed in standing position for the lateral view:

B3.1 Along the trunk 0-1-2-3

B3.2 Crossed on the chest 0-1-2-3

B3.3 Reposed at special support 0-1-2-3

B3.4 Other…. 0-1-2-3

# B4. cassette size

B4.1 Long cassette (80-90 cm long) 0-1-2-3

B4.2 Standard cassette (35-40-43 cm long) 0-1-2-3

B4.3 Small sizes (which?) 0-1-2-3

# B5. view

B5.1 Antero-Posterior 0-1-2-3

B5.2 Postero-Anterior 0-1-2-3

B5.3 Lateral at the beginning of treatment 0-1-2-3

B5.4 Lateral at the final visit 0-1-2-3

B5.5 Lateral at regular interval 0-1-2-3

B6. special views:

B6.1 oblique “plan d’election” of Stagnara 0-1-2-3

B6.2 side bending 0-1-2-3

B6.3 supine traction 0-1-2-3

B6.4 axial for rib hump 0-1-2-3

B6.5 left hand for bone age (Greulich-Pyle) 0-1-2-3

B6.6 others …

**B7. radiation protection** B7.1 Gonads 0-1-2-3

B7.2 Breast 0-1-2-3

B7.3 Thyroid 0-1-2-3

B7.4 Others… 0-1-2-3

**B7.4** “The first radiograph usually is taken without any radiation protection thus any skeleton anomaly could be detected” 0-1-2-3

B8. Parameters for systematic use:

B8.1 Cobb angle 0-1-2-3

B8.2 Fergusson angle 0-1-2-3

B8.3 Others:

B8.4 C7 shift 0-1-2-3

B8.5 Apical vertebra transposition 0-1-2-3

B8.6 Others:

Axial rotation of vertebra:

B8.7 Nash and Moe grades 0-1-2-3

B8.8 Perdriolle 0-1-2-3

B8.9 Drerup 0-1-2-3

B8.10 Raimondi 0-1-2-3

B8.11 Mehta rib-vertebra angle (RVA apical) 0-1-2-3

B8.12 Segmental RVAs 0-1-2-3

B8.13 Others ……

Sagittal

B8.14 Kyphosis Th4(5)-Th12 (sagittal Cobb) 0-1-2-3

B8.15 Lordosis angle L1-L5 (sagittal Cobb) 0-1-2-3

B8.16 Lumbo-sacral angle (L5-S1) 0-1-2-3

B8.17 Double rib contour sign 0-1-2-3

B8.18 Others for sagittal plane ….

B8.19 Sacral slope 0-1-2-3

B8.20 Pelvic incidence 0-1-2-3

B8.21 Risser sign 0-1-2-3

B8.22 Triradiate cartilage stage (open - closed) 0-1-2-3

B8.23 Others for bone age… 0-1-2-3

B9. Radiological measurements are made by:

B9.1 Myself (the treating person) 0-1-2-3

B9.2 Radiologist 0-1-2-3

B9.3 Other person… 0-1-2-3

B10. Where the X-ray examination is performed:

B10.1 At any X-ray office of patient’s choice 0-1-2-3

B10.1 At indicated X-ray office only 0-1-2-3

# B11. Schedule for X-ray exam

B11.1 First visit:

B11.2 Always 0-1-2-3

B11.3 Only if clinically suspected 0-1-2-3

Interval among subsequent X-rays during observation:

B11.5 3 months 0-1-2-3

B11.6 6 months 0-1-2-3

B11.7 12 months 0-1-2-3

B11.8 Other 0-1-2-3

Interval among subsequent X-rays during management with physiotherapy:

B11.9 3 months 0-1-2-3

B11.10 6 months 0-1-2-3

B11.11 12 months 0-1-2-3

B11.12 Other 0-1-2-3

Interval among subsequent X-rays during brace treatment:

B11.13 3 months 0-1-2-3

B11.14 6 months 0-1-2-3

B11.15 12 months 0-1-2-3

B11.16 Other 0-1-2-3

B12. Type of radiograph during brace treatment:

B12.1 In brace radiograph 0-1-2-3

B12.2 Out of brace radiograph 0-1-2-3

B13. What is the follow-up for the final X-ray (outcome of treatment):

B12.3 1 year after completion of treatment 0-1-2-3

B12.4 2 years after 0-1-2-3

B12.5 Other

Comments concerning radiographic exam:

## Part C. Surface topography measurements

**C1. Hardware** Formetric

Another raster stereographic

ISIS

Quantec

Moire

Auscan, Goals

Surphaser

Other

C2. What physical phenomenon the hardware you use is based on:

C2.1 Projection of a grid on the body surface and light interferention (Moire)

C2.2 Projection of digitized grid and then digitized image capture with automatic analysis by software (Formetric, other raster stereography)

C2.3 Projection of a light bundle moving along parallel lines and automatic image analysis (ISIS)

C2.4 other…

**C3. Position of the patient** C3.1 Standing upright 0-1-2-3

C3.2 Standing forward bent 0-1-2-3

C3.3 Sitting upright 0-1-2-3

C3.4 Sitting forward bent 0-1-2-3

C3.5 Other 0-1-2-3

**C4. View** C4.1 Back 0-1-2-3

C4.2 Front 0-1-2-3

C4.3 Other 0-1-2-3

# C5. Surface topography hardware available

C5.1 At the place of my practice 0-1-2-3

C5.2 At proximity, easily accessible 0-1-2-3 C5.3 Not available 0-1-2-3

C6. Surface topography examination (patient’s positioning, skin markers etc.) is done by:

C6.1 Myself (the treating person) 0-1-2-3

C6.2 Physiotherapist 0-1-2-3

C6.3 Nurse 0-1-2-3

C6.4 Technician 0-1-2-3

C6.5 Other 0-1-2-3

# C7. Surface topography measurements

**(choosing points, drawing lines at the computer screen etc. ) are done by:**

C7.1 Myself (the treating person) 0-1-2-3

C7.2 Physiotherapist 0-1-2-3

C7.3 Nurse 0-1-2-3

C7.4 Technician 0-1-2-3

C7.5 Other 0-1-2-3

C8. You make interpretation of your surface topography exam basically on:

C8.1 Image created by the software 0-1-2-3

C8.2 Values of parameters calculated by the software 0-1-2-3

**C9.** Anatomic landmarks which should be systematically taken into consideration:

C9.1 Spinous processes 0-1-2-3

C9.2 Posterior iliac spines 0-1-2-3

C9.3 Rib prominence (rib hump) 0-1-2-3

C9.4 Occiput 0-1-2-3

C9.5 Neck 0-1-2-3

C9.6 Shoulders 0-1-2-3

C9.7 Scapulae 0-1-2-3

C9.8 Waist 0-1-2-3

C9.9 Coccyx 0-1-2-3

C9.10 Others 0-1-2-3

C10. Surface topography parameters (your recommendation for systematic use):

General:

C10.1 Spine length (curve line) 0-1-2-3

C10.2 Spine height (C7-S1 straight distance) 0-1-2-3

Body axis definition:

C10.3 Analogous to radiological vertical central sacral line 0-1-2-3

C10.4 C7-S1 line 0-1-2-3

C10.5 Other

C10.6 Measurements of main curve only 0-1-2-3

C10.7 Measurements of main and secondary curves 0-1-2-3

Frontal plane analysis:

C10.8 C7 shift 0-1-2-3

C10.9 Curve angle 0-1-2-3

C10.10 Apex distance from the body axis 0-1-2-3

C10.11 Other

Body asymmetry in the frontal plane:

C10.12 Shoulders 0-1-2-3

C10.13 Scapulae 0-1-2-3

C10.14 Waist 0-1-2-3

Special indices:

C10.15 Weiss index 0-1-2-3

C10.16 POTSI index 0-1-2-3

C10.17 others

**C11. Sagittal plane analysis:**

C11.1 C7 relation to S1 0-1-2-3

C11.2 Cervical lordosis 0-1-2-3

C11.3 Thoracic kyphosis 0-1-2-3

C11.4 Lumbar lordosis 0-1-2-3

The limits (vertebral levels) for measuring thoracic kyphosis

and lumbar lordosis, are they :

C11.5 Automatically indicated by the software 0-1-2-3

C11.6 Manually indicated 0-1-2-3

C11.7 Segmental analysis of the profile available 0-1-2-3

C11.8 Segmental analysis of the profile desirable 0-1-2-3

Other

**C12. Transverse plane analysis:**

C12.1 Trunk rotation in main curve 0-1-2-3

C12.2 Trunk rotation in compensatory curves 0-1-2-3

Special indices:

C12.3 HUMP SUM 0-1-2-3

C12.4 DAPI index 0-1-2-3 C12.5 Others

The level to measure trunk rotation is indicated:

C12.6 Automatically by the software 0-1-2-3

C12.7 Manually by the person analyzing data 0-1-2-3

C12.8 other

**C13. Pelvis:**

C13.1 Posterior superior iliac spines height 0-1-2-3

C13.2 Posterior superior iliac spines depth

(difference in the distance from the camera) 0-1-2-3

C13.3 Pelvis position corrected before examination

to achieve iliac spines level at height and depth 0-1-2-3

Other comments on surface topography:

*Thank you for providing your opinion and your experience !*

The answers to the questionnaire will be reported as pooled data only, i.e., the individual respondent's answers will be kept confidential.

**Questionnaire respondent’s demographics**

Name: ……………………………………………………

Surname: …………………………………………

Specialty: ………………………………………

Professon

I am working under the:

1. o National (public) health system
2. o Private sector

Institution’s name: …………………………...

I’m working:

1. o In team with…………………………...
2. o Alone

How many patients with scoliosis do you evaluate each
working week ? 1 or less 1-5 6-10 11 or more

Address: ………………………………………………

Country/State: ……………………………….

e-mail: ………………………………………………..

Fax: ……………………………… Tel: ………………………………………………..

If not returned during the Consensus Session, please send the questionnaire to:

kotwicki@ump.edu.pl
