# Supplementary material for: Methodology of evaluation of morphology of the spine and the trunk in idiopathic scoliosis and other spinal deformities - 6th SOSORT consensus paper
Source: Scoliosis. 2009 Nov 26;4:26. doi: 10.1186/1748-7161-4-26 (PMC2794256; doi:10.1186/1748-7161-4-26)
Supplement: Additional file 3 — Proposal for SOSORT Scoliosis Evaluation Form. evaluation form for scoliosis recommended by the authors. [file 1748-7161-4-26-S3.DOC]

*Proposal for SOSORT Scoliosis Evaluation Form*

Date ___________ Number ____________

Name ___________________________________ Sex F M Date of birth ____________

………………………………………………………………………………………………………

Diagnosis ____________________________________________________________________________________________________

Lenke type _____ Rigo type ______ King type _______

……………………………………………………………………………………………………….

Height ____ cm Sitting height ____ cm Weight ____ kg

Date of menarche _________

Tanner: breast __ pubis __

Lower limb discrepancy Y / N : shorter L / R __cm

Clinical exam with compensation? Yes / No

……………………………………………………………………………………………………….

Scoliometer _____ ° _____ ° _____ ° position standing / sitting

prox.Th main Th Th-L / L

C7 plumb line centered / right ____cm / left ____cm

……………………………………………………………………………………………………………………………………………..

TRACE index _____

………………………………………………………………………………………………………………………………………………..

Cobb angle _____° _____° _____° Risser sign ____

prox.Th main Th Th-L / L

C7 shift centered / right ____cm / left ____cm

Apical vertebra rotation _____° _____° _____°

(Perdriolle) prox.Th main Th Th-L / L

Standing lateral radiograph

| Parameter | Th2-Th5 Cobb | Th5-Th12 Cobb | Th10-L2 Cobb | L1-L5  Cobb | Sacral slope | Incidence |
| --- | --- | --- | --- | --- | --- | --- |
| value | ___° | ___° | ___° | ___° | ___° | ___° |

.............................................................................................................

Surface topography

Thoracic kyphosis ____ lumbar lordosis ____ POTSI ______

Trunk rotation _____° _____° _____°

prox.Th main Th Th-L / L

………………………………………………………………………………………………………………………………..

Legend:

Tanner – for pre-menarchial girls older than 10 ys.

Lower limb discrepancy – assessed with ASIS position, checked with PSIS position.

Radiograph – standing, spontaneous posture, PA or AP, long cassette preferable.

C7 shift is measured to the Vertical Central Sacral Line
